# Plastic Filling, and the Basal Principles of the New Departure

**Published:** 1878-05

**Authors:** J. Foster Flagg

**Affiliations:** Philadelphia.


### AMERICAN JOURNAL

OF

### DENTAL SCIENCE.

Vol. XII. THIRD SERIES—MAY, 1878. No. 1.

### ARTICLE I.

### *Plastic Filling, and the Basal Principles of the New Departure.*—Continued.

BY J. FOSTER FLAGG, D. D. S., OF PHILADELPHIA.

Article II. Accepted Creed.—*Some have faith in contour, others have faith in separation.*

Article II. New Departure.— *We do not believe that either has much to do with the final result.*

For years the discussion between contour and separation has gone on. With Arthur, Bonwill and Chupein for the A, B, C of the "separationists," and Atkinson and Webb as representatives of the whole alphabetic list of "contourists," no definite decision has yet been reached. Now it seems to us that if either was, in truth, decidedly the right way, it would have been found out by this time.

In regard to this contest *we* have been lookers-on, and our statistics do not yet show us which is best. Contour fillings fail, and fillings in separations fail; and when the contours fail it is said, "It ought to have been separated;" and when the fillings in the separations fail, it is said, "The separation is not made rightly." Meanwhile the teeth decay.

For me, in a strong, solid tooth, I would, for durability, just as soon make a contour filling as I would a separation; but on general principles and for comfort I prefer to give my patients the benefit of what is called "contour." My experience is that it is better than the separation—certainly on the score of comfort, and probably on the score of durability.

ARTICLE III. ACCEPTED CREED.—*Failure in operations is mainly due to defective manipulation.*

ARTICLE III. NEW DEPARTURE.—*Failure in operations is mainly due to incompatibility of filling material with tooth bone.*

Gentlemen, all the elegant fillers—high style, $75, $150, $500 plug men—tell you that failures are due to defective manipulation. Now, gentlemen, if this is so, why don't you manipulate better? Simply because you cannot. And is it nothing against gold that men cannot manipulate it? Is it nothing that this has been proved by an experience of more than half a century? The record of to-day is not better than the record of fifty years ago. You talk about the "finer work," the "improvement in instrumentation," the "blessed rubber dam;" but with all these adjuncts, and while you have put all these gifts to tests which have proved their greatest capability, your results are: larger fillings, more difficult fillings, more tedious fillings, more painful fillings, more expensive fillings, less compensating fillings (both to yourselves and patients,) and *more decided failures.* I state this squarely. For every *one* of these large, difficult, tedious, painful and expensive fillings that have given a dozen years of service, I think you will acknowledge that I am within bounds when I say *a dozen* have proved failures.

My statistics show that the average of gold failures, for the last year, is greater than for any previous year. 100

gold failures were classified upon my tables last year, for the whole twelve months; and this year, up to this month, I have already 86, making a total, for 1877, of 103—many of them far worse failures. This is the truth. This shows what attempts are being made and what failures are the results.

It was only on last Friday morning that I renewed (by guarding) a gold filling, which had been introduced *less than three years* before in a cavity upon the *mesial face of the right upper second bicuspid, with the first bicuspid out.* I want you to think of this, and note the exceeding difficulty (?) of the original operation. The patient J. W., one of our largest car wheel manufacturers, told me he paid $20 for the filling; and I showed him that I could pass a broad, thin instrument deep into the crevice between the filling and the tooth at almost any point. I told him that *I could not say* of such beautiful work what I believed the gentleman who did it would say of it, viz.: that it must have been *defective in manipulation;* but that I knew just how soft his teeth were (he had formerly been my patient, had left to see if he could obtain better results than I had been able to make for him, *and had returned*,) and that I believed the failure was due to *incompatibility of gold with tooth bone.* He said he thought $7 a year was a little too much to pay for one tooth, and then I, naturally (for I am only human,) expatiated to him as I am doing to-night to you. I said, "Suppose, Mr. W., that my filling lasts for only three years. It will take me an hour to introduce and finish it, and I shall charge $5. Your former operator charged you $20, because it took him four hours. His work has proved dear, but mine cannot be called cheap, because it returns me the same as his does to him. It may be cheap to you." To which he replied: "Doctor, your argument is just that which the cast iron wheel men use against the steel wheel men, and we consider it solid."

We believe that "failure in operations is mainly due to incompatibility of filling material with tooth bone." This again opens all the question upon which rests our vitality. When I tell you that, *experimentally*, the contact of gold and bone, amalgam and bone, tin and bone, and gutta-percha and bone, results, again and again, in greatest loss of substance in that bone which is in apposition with the *gold*, I say, "He that hath eyes to see, let him see; and he that hath ears to hear, let him hear." And when I see just the same thing occurring in the mouth every day that I practice, and then read that "experiments out of the mouth must be taken for nothing," I cannot but feel that such writers are "straining at gnats," while their patients are "swallowing camels."

Article IV. Accepted Creed.—*A tooth that is worth filling at all, is worth filling with gold.*

Article IV. New Departure.—*A tooth that can be so treated as to be satisfactorily filled with anything, is worth filling.*

The fourth article of the accepted creed and its twin brother, "a tooth that can be filled at all, can be filled with gold," seem to me such perfect puerility that I have no patience with them. This latter enunciation is from our good friend Taft's last work on Dentistry—"up to the present status of the profession," the preface says.

The scathing criticism of this production in the *Cosmos* induced me to examine more closely than I might otherwise have done this effort of Dr. Taft's. Among things like "vacuums" between pulp-caps and pulps, I found this statement, that "a tooth that is worth filling at all, is worth filling with gold." But it is not the best, the most acceptable, the most expeditious, or the most comfortable way to do. It is not the most *serviceable* way to do. I assert that experience proves this; but beyond that, I most emphatically deny both assertions. I deny that "a tooth which cannot be serviceably filled with gold, is not worth filling." I deny that "any tooth which can be filled at all, can be filled with gold." No one, not even the most expert, can fill with gold every tooth that can be filled. How much less, then, can the average respectable practitioner of dentistry do this thing? You may get gold into it, after a fashion—but you cannot fill it, according to your own ideal of what a filling should be.

On the other hand, with a plastic filling material it can be filled, and filled nicely, and so as to be comfortable and serviceable for many years. Experience has shown this. The profession, and the coming text-books, when they are truly "up to the status of the profession," will admit it. Therefore we say that "a tooth that can be so treated as to be *satisfactorily* filled with *anything*, is worth filling."

Article V. Accepted Creed.—*Unskilled and unscrupulous dentists fill with tin covered with gold, thereby causing galvanic action, pulpitis, death of the pulp, abscess, and loss of the tooth.*

Article V. New Departure.—*Skillful and scrupulous dentists fill with tin covered with gold, thereby preventing decay and pulpitis, and thereby saving the tooth.*

Experience had proved that tin would save teeth which gold did not. The accepted belief was, *and is*, that it did so because of its softness and ease of manipulation (for which qualities, by the way, we have never heard it decried.) *Our* belief now is that it saves teeth because of its *low conductivity*, and because *it is itself attacked.* Experience had also proved that the tin wore out; and so, the happy combination which would save the tooth and yet not wear out having been devised, it was decried and defamed as "*unskillful* and *unscrupulous.*"

Fifteen years ago this was the accepted practice of my office. It is because of this principle that the operation of "guarding" with a crescentic guard of tin, amalgam or gutta-percha at the "vulnerable spot" has retained failing gold plugs by the hundred for continued usefulness. I, myself, had, up to April, 1877, 1,055 gold plugs, of my own and other operators, doing service *only* because they had been "guarded" in this way. Gold doing well because it was *taken care of* by *tin*, *gutta-percha* or *amalgam*—a pretty picture!

ARTICLE VI. ACCEPTED CREED.—*A filling, to be good must not leak.*

ARTICLE VI. NEW DEPARTURE.—*A filling may be the best that is known for the tooth, and yet leak badly.*

How do we maintain this point? We hold that this is true because of the fact that gutta-percha makes a leaky filling. See this filling in this tube; every care was taken that it should not leak. It was packed even better than it could have been in a tooth, and yet, as you see, it leaks badly. But you will say that a glass tube is not analogous to a tooth. I am prepared for that. Here are small cups of ivory. This material is almost identical with that of tooth bone. These were filled, as you see, the one with red gutta-percha and the other with Johnstons' Premium Stopping; and yet both leak.

*Why* do we maintain it? We do so from the fact that *we wish to make it a basal principle, that mere leakage, itself, is not the thing* which makes a filling capable or incapable of *saving a tooth.* It depends upon what material leaks, and what it leaks in contact with. If a leak of vitiated saliva occurs between tooth bone and a material which is a *good conductor, and is itself unattacked*, then that leaky filling is very bad. But if even vitiated saliva leaks between tooth bone and a material which is, practically, a non-conductor, and which has a surface that is *neither bright nor oxidizable*, then leakage is *proved* by *experience* to be *not* practically detrimental. This we hold to be the reason why gold fillings "eat themselves out" of soft teeth in from two to five years; and why gutta-percha remains untouched (except by attrition,) and preserves such teeth for many, many years.

ARTICLE VII. ACCEPTED CREED.—*Gutta-percha, properly used, is good enough for temporary fillings.*

ARTICLE VII. NEW DEPARTURE.—*Gutta-percha, properly used, is the most permanent filling material we possess.*

When my friend, Dr. Hawes, was in my office for a part of two days, some months ago, I operated before him. Among other patients was a young lady from Orange, New Jersey. I showed him a line of several gutta percha fillings, in front teeth, which had been introduced seven years before. I showed him a gutta-percha filling in the buccal face of a left lower molar, which he thought was "somewhat cupped" and would "hardly pass for a permanent filling." The patient informed him that it had been doing service for *twenty* or *twenty-one* years, and I added that if it lasted as much longer, I should mark it down as permanent!

Twenty-six years ago Dr. G., of Philadelphia, came to my father, desiring his services. My father introduced eleven gold fillings—good, old-fashioned, solid, "straight from the shoulder" fillings. Besides these cavities, there were four enormous places in the four *wisdom-teeth* (poor teeth, according to accepted views,) and these were filled, "experimentally," three with amalgam and one (the buccal face of the right lower wisdom) with gutta-percha, with the idea that they might "last for a year or two." The last one of the eleven gold fillings was renewed a few months since, and the three amalgam fillings, *and the gutta-percha filling*, are still preserving their forlorn and worthless wisdom-teeth, as they have done for *more than a quarter of a century.*

These are not isolated cases. They are, far more, *typical* cases. I have, in the mouths of my patients, hundreds upon hundreds of gutta-percha fillings, which have been doing service for from five to fifteen years, and bid fair to continue so doing for years to come.

Gentlemen, I feel, when I introduce a gutta-percha filling into a large cavity on the buccal, mesial or distal face of a tooth—close to the gum, with frail walls, and almost into the pulp—that I have (if it be possible) saved that pulp. I feel that I have placed there a material which, even if it be gradually softened and worn out, will save the tooth until the last thin stratum, only, still remains. I feel that if it wears, an easy, inexpensive reparation can be effected by the addition of a little more of the same material at any time. I feel that I have done my patient that real service which he or she had hoped to have bestowed.

This is why we say that "gutta-percha, properly used, is the most permanent filling material we possess.

ARTICLE VIII. ACCEPTED CREED.—*A good gutta-percha filling, in its proper place, is better than a poor gold one.*

ARTICLE VIII. NEW DEPARTURE.—*A poor gutta-percha filling in its proper place, is better than a good gold one.*

Some time ago a few of our Philadelphia gentlemen saw themselves on paper as saying almost too much in favor of gutta-percha; so they corrected it by publishing that they did not mean to say that gutta-percha was as good as gold; what they meant was, "that a real good gutta-percha filling, *in its proper place*, was better than a *poor* gold one." Mr. J. G. W. had a gold filling introduced upon the mesial face of the left upper lateral, by one of our best operators. In about two years the filling failed. It was renewed, and in less than two years this second filling failed. We think we have a right to infer that both these fillings were good ones. Upon the occasion of a third visit, a gutta-percha filling was hastily introduced, to serve merely, *as was stated*, a temporary purpose. While yet the temporary filling was in place, the gentleman's partner (a patient of mine) induced him to call upon me. I was told the story. I examined the gutta-percha filling. It bore evidence of its hasty insertion, having overhanging edges. These I trimmed with a warm instrument, and asked him to give me the first proof of his confidence in his partner's recommendation and my judgment, by allowing the gutta-percha filling to remain, as it was a proper place for it. Already that filling has lasted longer than either of the gold ones, and, from appearances, it will last far longer than both the gold ones together.

This is also not an isolated case, and it is from such instances that we deduce "that a *poor* gutta-percha filling, *in its proper place*, is *better* than a *good* gold one!"

ARTICLE IX. ACCEPTED CREED.—*Amalgam, per se, is a poor filling material.*

ARTICLE IX. NEW DEPARTURE.—*Amalgam, per se, is an excellent filling material.*

I do not mean to say that all you gentlemen think or say that amalgam is a poor filling material. But I do mean that this is the teaching of the most recent and accepted text-books and authorities Read Taft, pages 93, 94 and 95, and you will see the "creed" of "Ann Arbor" and "Ohio."

I do not wish you to think, for a moment, that we regard amalgam as the *ultima thule.* By no means. While we recognize its great excellence, we also recognize its great deficiencies.

The relative durability of the three materials—gutta-percha, amalgam and gold,—is a question which I have been engaged upon for more than fifteen years; during which period I have endeavored to tabulate this matter by following the career of over *twenty thousand* fillings. Of course I recognize the impossibility of doing this with scientific accuracy. I admit that my result is merely a guess—but, gentlemen, I regard it as a *very close guess*—that, as the result of *average dentistry*, gold fails in fifteen years in 71 per cent. of its cases, and amalgam in 54 per cent. of its cases.

Understand me, this is *average* dentistry. It is not *poor* dentistry, for the gold efforts of such, you know, would not nearly reach such a standard. Neither is it truly "first-class" dentistry. If it were, I should be ashamed of first-class dentistry. But it is very close to what our profession, as a profession, is doing for those of humanity at large who come to us for services.

Now, when you reflect that all the good places, all the easy places, all the accessible places, as a rule (admitting all exceptions,) are filled with gold; and that all the bad places, all the hard places, all the "forlornities," as a rule (admitting the exceptions,) are filled with amalgam, *we* think that it does not tell so badly for amalgam; while we say for the gold, that it is a pity it is so "incompatible with tooth bone."

For gutta-percha, with 2,000 fillings of an average duration of eight years (these were in carefully-selected places, but all in *soft* teeth,) the failure is but eight per cent.—160 failures, in soft teeth, in 2,000 cases, in eight years. These failures were mostly from disintegration of the gutta-percha. The action of the fluids of some mouths causes this material to soften, puff up and disintegrate. In such mouths gold is almost worthless for the average practitioner, except upon the articulating faces of the teeth; and in these mouths tin and amalgam have proven themselves our most reliable filling materials.

But you will say that gutta-percha fillings do not ordinarily last that way. That is because they are put in, definitely, only as temporary work, being removed in a short time to be replaced with work which is *called* permanent. The gutta-percha is almost always found (no matter how long it has been allowed to remain) *preserving the tooth* perfectly; and is frequently complimented by the operator for having done so, even while he is introducing his permanent filling, which, in truth, has often to be renewed sooner than would have been the case had he allowed his temporary filling to have remained.

Again, your instructions as to working gutta-percha, from the direction to "heat it on a porcelain or metal slab over a spirit lamp" (there is no surer method of ruining the material from over-heating) to the "holding of an instrument on the filling and pressing it till it is cool"—these instructions, I think, amount to nothing. Take a cavity in a lower second molar, first molar and wisdom-tooth in place. The cavity shall begin on the mesial face and deeply circumscribe the tooth, close to and partially under the gum, until it has passed along the buccal and distal faces and impinged a little on the lingual face. There is a place to fill! You all have seen just such places, and they are excellent to fill with gutta-percha.

I have often listened to our good friend Atkinson, as with streams of intelligence scintillating from his flashing eyes, and his whole countenance beaming with enthusiasm, he would expatiate on the wonderful workings of the mallet in the "hands of intelligence." But I tell you, gentlemen, it would take no less than *four instruments* in the hands of *four intelligences* to press in such a plug *till it got cold!*

ARTICLE X. ACCEPTED CREED.—*The use of plastic filling materials tends to lower the standard of dentistry, thereby diminishing its sphere of usefulness.*

ARTICLE X. NEW DEPARTURE.—*The use of plastic filling materials tends to lower that dentistry which has for its standard of excellence ability to make gold fillings, but very much extends the sphere of usefulness of that dentistry which has for its standard of excellence ability to save teeth.*

This is where we rest our case. It is for the *salvation of teeth* that I have come to speak to you to-night. I have spoken only with that end in view.

I don't wish to speak of good, strong teeth. I don't wish, *at this time*, to tell you of my practice of to-day—what I am experimenting on, what I am tabulating. I have only to tell you what I have done, and what conclusions I have arrived at as the result of experience and long years of practice.

I do not wish to say anything to you of the teeth which you are in the habit of filling successfully, and, as we express it, satisfactorily, with gold; teeth of a dense structure, whose cavities have walls so strong that you can impact a filling which lasts a life-time. But I do ask that you will gradually discontinue this packing of gold into teeth that are so poor, so frail, so unsubstantial, that it is, to say the least, doubtful whether the result will be creditable to your profession, or satisfactory to your patients. Remember, "$7 a year seems to me too much to pay for one tooth."

Commence, if you will, with those poorer and more miserable teeth which you would condemn to the forceps, and fill them with plastic fillings.

You will soon be agreeably surprised with your results in the new direction. You will soon find that much that has been told you by tradition and text-books has no *reality* in it. It is true that our results are produced more easily, comfortably and economically to the patients, but they will not complain of that. It is true that our results are produced with greater ease to the operator, and, for this reason, it is argued by our opponents that we do it from "laziness."

But I tell you that it is not from laziness. It is from a desire to lay aside the forceps. It is from a desire that our patients shall eat upon teeth the roots of which, at least, are in their jaws. It is from a desire that they shall be exempt from the infliction of artificial work. It is from a desire to extend the blessings which the hand of our profession holds to bestow. It is that we may be sought rather than avoided, that we may be extolled rather than decried, that we may be esteemed rather than censured, that rather than be feared, we may be loved and *respected.*

It is for this that I have worked; and blessed be God that he has so sustained and directed me that I have, every day, fresh cause to believe that I have gained the love of my patients and the respect of my professional brethren!

And now I find on the programme "Incidents of Practice." I suppose it meant "incidents of *my* practice," and so I have come prepared with a few to offer you.

Since twelve years ago (July, 1865) I have been keeping the money record of five sets of teeth, among the softest and frailest of those under my charge.

These were poor enough; decayed through and through, so that you could *string* five or six in a row; broken off, decayed down to and under the gum. From 1865 to 1877

(twelve years) these teeth have been kept comfortably in order. Not one tooth has been lost from the five mouths during all this time. That alone is something. But in adding up the aggregate of all their bills, and dividing the total by five, it shows that those mouths have been kept in order for less than $15 each per year ($14.80.) This brings dentistry within the reach of many who have heretofore been deprived of its benefits. It brings it where it belongs —to be a blessing to the millions; and this is why we say that our practice "very much extends the sphere of usefulness of that dentistry which has for its standard of excellence ability to save teeth."

Prof. J. G. Richardson had in 1869 a right upper lateral, right upper central and left upper central decayed and needed saving. The lateral and left central were filled with gold; but the right central, being much worse than either of the others, my advice was asked in regard to it before it was filled. I said it would be best to fill it with gutta-percha. "What! a front tooth?" was the reply. "Why, yes! why not?" said I; "the man wants to save his front tooth just as much as he would a back one!" So it was filled with gutta-percha. In a few months from that time the lateral ached so badly during an entire night, that in the morning (I think it was on Sunday—I was not in the city,) he called upon a dentist and insisted that it should be extracted.

In the course of time an ominous shadow passed over the left central, which told that its pulp had quietly died a natural death. It was entered, the pulp removed, and it is now doing good service as a pulpless tooth. But the worst of the three, the one that in 1869 most *needed* saving *has been saved*, and is, to-day, a living, healthy, brilliant tooth. I saw it but a few days since. The filling is a little worn. I have an appointment with him, to add a little gutta-percha to it, on my return, when, with fifteen minutes' work, I shall start it off for another eight or ten years' journey.

Mrs. McG., left upper lateral; in this tooth two gold fillings, one $5, the other $6, lasted fifteen years. By the failure of both, the tooth had become pretty badly decayed. *Twelve* years ago a gutta-percha filling was introduced for $4. With two trifling repairs, so trifling as to cost nothing, that filling has preserved the tooth perfectly.

Mrs. B., eleven years ago, had a range of gutta-percha fillings introduced by a gentleman in Germantown. Her teeth were very frail. The fillings cost $25. In about two years, while visiting one of our Philadelphia gentlemen, she mentioned that it was two years since had had any work done. She requested an examination, and was told, *very emphatically*, that there was some great mistake, that there was nothing but *temporary work* in her mouth, and that *she* ought not to have any other than the *very best* which was *gold work.* All the gutta-percha fillings were removed and replaced with gold, except one poor molar, which was stated to be "worth nothing except gutta-percha." In about two years from that time she visited my office at the recommendation of a friend, when I found every gold filling in such condition that my friend, Dr. Dixon, would have thought proper to have them removed. I said to her that the mistake had been made of filling her teeth with *gold*, when they ought to have been filled with *gutta-percha;* and, showing her the *poor molar*, said, "Do you see, this poorest of all the teeth has been filled with gutta-percha, and it is the only one that is not very defective."

Her eyes filled with tears as she told me the story of her four years' experience, and, as she finished, she asked what she should do. I suggested that she should call on and show the gentleman who did the gold work, the result of his labors. This proposition being declined with a show of considerable spirit, I then suggested that she should let the first gentleman replace the defective fillings with gutta-percha, as he had had the good judgment to do in the first place. She said that he was in Florida and would not be back until some months later, and then asked if I would fill them with gutta-percha. I did so; and some three years after (she had gone to the far West) I heard that all was comfortable and doing well.

Just $200 for $25 worth of work, as one practical experience of gold fillings in soft teeth!

Dr. S. has three daughters. The eldest had centrals and laterals filled with gold at nine years of age. These fillings failed and were renewed when she was eleven. Again they failed, and at thirteen and fourteen years of age they were severally renewed. Under these fillings the pulps died, and the teeth came to me for treatment. She has four discolored pulpless teeth.

The teeth (centrals and laterals) of the second daughter were filled when she was nine, with malleted fillings. At eleven they failed, when hand pressure fillings were introduced. These failed when she was between thirteen and fourteen, and gutta-percha fillings were then introduced. At sixteen these are good, and all the pulps are vital.

The centrals and laterals of the third daughter were filled with gutta-percha when she was nine. She is now twelve, and all the fillings are just as they were when introduced.

These are typical cases of my gutta-percha results. Now for amalgam.

Mr. D., the great umbrella manufacturer of our city, came to me in 1859 with a left upper molar. It had been pronounced unworthy of salvation, and he desired ether as an anæsthetic. I suggested that it would be a very great loss to him, and proposed treating it and building on an amalgam crown. This was done, and to-day he calls it his "veteran;" and it has enlisted many a new recruit into my army of teeth "not worth saving!"

Mr. C. W. came to me, in 1857, with no teeth to spare. He was in young middle life (twenty-seven or eight,) and had already lost many teeth. The tooth he came to have extracted was filled then with amalgam. He broke the crown off a *few weeks since.* I show it to you here. The roots have a full amalgam crown built on them now.

Since 1857 I have a list of a few over 500 patients who have come to me, having lost already what teeth they could comfortably spare; and from all these mouths, since that time, *not one tooth has been extracted.* Can any of you do any better than that with your $40 gold fillings?

Now I have here three teeth which I have brought you on the *discoloration* question. I would have brought more, but *unfortunately* the patients are eating on them! This one was in service 15 years; this one for 17 years; and this one for 22 years. Do you see that these teeth are *not one particle discolored?* These fillings are made of Townsend's Amalgam, which contains only tin and silver. When such fillings leak sufficiently the tooth becomes discolored,—just as teeth become discolored when gold fillings leak; but so long as they are tight, and in teeth of average quality as to structure, then discoloration is the *rare exception*, rather than the rule. I tell you, let an *average* operator introduce into one hundred *average* teeth fifty fillings of gold and fifty fillings of amalgam, and in ten years there will not only be more of the amalgam fillings doing good service, but among the fillings that are left there will be found more discoloration around the gold fillings than around those of amalgam. This is a startling statement, at least to some of you; but I assure you that I do not make it loosely, but as the result of long and careful watching—as the result of many years of patient observation.

Again, your ideas of amalgam are based almost exclusively upon its workings in the enormous cavities of almost completely abandoned teeth. You select teeth that you say are "worthy of nothing else." Now, is this fair? For if amalgam will save such teeth *even doubtfully well and exceptionally*, is it not worthy a trial in some of the *moderately sized* cavities of teeth of *somewhat better structure*, where, even yet, gold *occasionally* fails?

It was said on one occasion, by my very good friend, Dr. Bogue, that he "viewed with admiration the pin-head cavities in centrals filled with amalgam by Dr. Clowes." Now I have viewed, I *think*, a great deal more frequently with not so much admiration, the *enormous* cavities in centrals which *had become so* (I say this advisedly) as the result of two or three consecutive gold fillings in each. For me, I would far rather have one of Dr. Clowes' "pin-head" amalgam fillings in one of my centrals, than to have one of those evidences of "first class ability," such as carried death to the pulps of both centrals and laterals of the young lady whose case I have related to you this evening.

Nor, gentlemen, is this any unusual story of "accepted' dentistry. Front teeth, *par excellence*, are filled and refilled with gold, as the *best* that can be done, until pulp after pulp dies, tooth after tooth becomes discolored and crumbles away, root after root is extracted, and plate after plate is inserted. This is *stereotyped practice*, and I defy contradiction of the statement.

Now, gentlemen, I want to tell you one thing more which has grown out of our long work on plastic fillings—that we have not come before you prepared for only a small struggle. We are ready for the fight. We have opened our batteries against the accepted dentistry of the day. We are an aggressive party, and we purpose *insisting* that our "basal principles" shall be thoroughly discussed. We purpose making it *respectable* that teeth shall be filled with materials which experience has shown to be able to save them; and we purpose making it *not respectable* to fill teeth with materials which experience proves daily to be "unequal to the emergency." We purpose that "seven dollars a year for one tooth" shall be regarded as too much by *dentists*, as well as by *patients*.

Gentlemen, I have brought up thirteen hundred patients from childhood; and from the whole thirteen hundred mouths but *five* permanent teeth have been extracted, and these five teeth I have as the trophies of my success—not to show that my "scalps" are many, but to glory that they are so few. I do not say this to you boastfully. I could not do that; for my heart is too full of earnest, deep-seated thankfulness that it has been given to me to go in that path which has led to so much comfort for so many of my fellowcreatures. But I must plead guilty to a feeling of intense satisfaction, as I think of the fact that every one of *mine* eats upon full unbroken arches, above and below!

Gentlemen, make a beginning with the teeth you would extract, and treat them. Make *that* dentistry! Fill them with plastic fillinsg; and if, in twenty years, your experience shall have been that which mine has been, you will thank that great and excellent dentist, Elisha Townsend, as I thank him, for having had the hardihood to break the trammels of tradition and authority, in declaring that "teeth could be saved with amalgam which *he* could not save with gold."—*Dental and Oral Science Magazine.*

---

